# Tyrosine hydroxylase and β_2_-adrenergic receptor expression in leukocytes of spontaneously hypertensive rats: putative peripheral markers of central sympathetic activity

**DOI:** 10.1590/1414-431X20209615

**Published:** 2020-10-30

**Authors:** L.M. Nisimura, P. Bousquet, F. Muccillo, E. Tibirica, L.R. Garzoni

**Affiliations:** 1 Laboratório de Inovações em Terapias, Ensino e Bioprodutos, Instituto Oswaldo Cruz, Fundação Oswaldo Cruz, Rio de Janeiro, RJ, Brasil; 2 Laboratório de Investigação Cardiovascular, Instituto Oswaldo Cruz, Fundação Oswaldo Cruz, Rio de Janeiro, RJ, Brasil; 3 Department of Pharmacology, Faculty of Medicine, University of Strasbourg, Strasbourg, France; 4 Instituto Nacional de Cardiologia, Ministério da Saúde, Rio de Janeiro, RJ, Brasil

**Keywords:** Brain stem, Sympathetic nervous system, Leukocytes, Spontaneously hypertensive rats, Clonidine

## Abstract

The sympathetic nervous system (SNS) plays a fundamental role in the pathophysiology of cardiovascular diseases, including primary arterial hypertension. In this study, we aimed to investigate whether the expression of the rate-limiting enzyme in catecholamine synthesis, tyrosine hydroxylase (TH), and the β_2_-adrenergic receptor (β_2_-AR) in immune cells from peripheral blood, reflect central SNS activity in spontaneously hypertensive rats (SHR). TH expression in the lower brainstem and adrenal glands and β_2_-AR expression in the lower brainstem were analyzed by western blot analyses. In the leukocytes, TH and β_2_-AR expression was evaluated by flow cytometry before and after chronic treatment with the centrally-acting sympathoinhibitory drug clonidine. Western blot analyses showed increased TH and β_2_-AR expression in the lower brainstem and increased TH in adrenal glands from SHR compared to normotensive Wistar Kyoto rats (WKY). Lower brainstem from SHR treated with clonidine presented reduced TH and β_2_-AR levels, and adrenal glands had decreased TH expression compared to SHR treated with vehicle. Flow cytometry showed that the percentage of leukocytes that express β_2_-AR is higher in SHR than in WKY. However, the percentage of leukocytes that expressed TH was higher in WKY than in SHR. Moreover, chronic treatment with clonidine normalized the levels of TH and β_2_-AR in leukocytes from SHR to similar levels of those of WKY. Our study demonstrated that the percentage of leukocytes expressing TH and β_2_-AR was altered in arterial hypertension and can be modulated by central sympathetic inhibition with clonidine treatment.

## Introduction

Cardiovascular diseases are the leading cause of mortality worldwide, representing approximately 31% of all-cause mortality (1). Primary hypertension is a multifactorial disease, and sympathetic nervous system (SNS) hyperactivity plays an important role in its pathophysiology (2).

Central sympathetic hyperactivity and the resulting activation of the sympathetic outflow to the blood vessels, the heart, and the kidneys is a hallmark in hypertensive patients (2) and in experimental models of hypertension (3). Moreover, the increase in sympathetic activity is a mechanism for both initiating and sustaining elevated blood pressure (2). Additionally, sympathetic overactivity increases cardiovascular risk through the development of left ventricular hypertrophy, contributing to the origin of ventricular arrhythmias and sudden death and reducing the uptake of glucose by skeletal muscle, which can result in insulin resistance and consequent hyperinsulinemia (4).

Consequently, the assessment of the systemic levels of sympathetic activity appears to be crucial in the management of primary hypertension, allowing for the identification of a subset of hypertensive patients presenting sympathetic overactivity. In these patients, the modulation of central sympathetic activity using centrally acting antihypertensive drugs could be especially beneficial. Human regional sympathetic activity can be studied by microneurography of postganglionic sympathetic efferences directed to the skeletal muscle vasculature and by noradrenaline spillover, which is a measurement of organ-specific catecholamine release into plasma, together with direct measurement of plasma catecholamines and spectral analysis of heart rate signals (5). However, these different approaches display important limitations and disadvantages, and some of them are not feasible in daily clinical practice (6).

Tyrosine hydroxylase (TH) is a rate-limiting enzyme in catecholamine synthesis and a key component of the central and peripheral SNS (7). The activation of presynaptic β_2_-adrenergic receptor (β_2_-AR) by catecholamines leads to noradrenaline release (8) and induces enhanced cardiac output and myocardial contractility, acting on the most abundant β-adrenergic receptor subtype in the heart, β_1_-AR. α_2_-Adrenergic receptors (inhibitory auto-receptors) are also present in the noradrenergic nerve endings and are involved in the regulation of noradrenaline release through a negative feedback mechanism mediated by the neurotransmitter itself (8). During sympathetic overactivity, which is the case of several cardiovascular diseases, including arterial hypertension and heart failure, both α- and β-adrenergic receptors are continuously activated. This results in β_1_-AR desensitization with alterations of down-stream mechanisms (9), resulting in further activation of the β_2_-AR. Regarding the desensitization of the β_2_-AR on human leukocytes, it has already been demonstrated that β-AR agonists induce a rapid desensitization of the receptor with an associated redistribution of the receptor into a cellular compartment to which catecholamines have limited access (10).

The interactions between the autonomic nervous system and the immune system are considered to be bidirectional. Primary and secondary lymphoid organs have rich sympathetic innervations that are responsible for the release of noradrenaline and other neurotransmitters (11). Catecholamines are produced by thymic lymphoid and non-lymphoid cells, including epithelial and endothelial cells, platelets, and macrophages (12,13). Moreover, lymphocytes produce catecholamines and express alpha and beta adrenergic receptors (14).

The presence of components of the sympathetic system in leukocytes has been related to central sympathetic hyperactivity (14,15). Furthermore, the detection of components of the sympathetic system in leukocytes has been proposed as a simple strategy to assess the levels of sympathetic activity of the central nervous system (CNS) in the periphery (14–16). Human and rat mononuclear cells express TH, and the catecholamines produced by these cells are capable of acting in an autocrine and/or paracrine manner (17).

The expression of β_2_-AR is higher in the mononuclear cells of hypertensive patients than in those of normotensive controls; moreover, there is a significant correlation between β_2_-AR density and mean arterial blood pressure in primary hypertension (15,18).

Thus, in the present study, we investigated the existence of an association between TH and β_2_-AR expression in leukocytes from peripheral blood with the plasma levels of catecholamines, blood pressure, and heart rate. Moreover, the chronic treatment of the animals with the centrally acting sympathoinhibitory drug clonidine was used to test the effects of the negative modulation of the central SNS on TH and β_2_-AR expression.

## Material and Methods

### Animals

Male Wistar Kyoto rats (WKY) (from the Animal Breeding Center, CECAL, Oswaldo Cruz Foundation, Rio de Janeiro, Brazil) and spontaneously hypertensive rats (SHR) of the Okamoto-Aoki strain (from the Central Animal Facility of State University of Campinas, Campinas, Brazil), aged 12 to 14 weeks and weighing 200-250 g, were used in this study. The animals were housed at a maximum of 4 individuals per cage, kept in a specific-pathogen-free (SPF) room at controlled conditions of light (12-h light/dark cycle) and temperature (22±1°C), and were provided sterilized water and standard rat chow *ad libitum*. The experimental procedures were approved by the Board of the Care and Use of Experimental Animals (CEUA, Oswaldo Cruz Foundation, license LW-46/13).

### Clonidine treatment

SHR aged between 12 and 14 weeks were treated with 0.1 mg·kg^-1^·day^-1^ clonidine (Sigma-Aldrich, USA) dissolved in distilled water once per day for 28 days by gavage. The clonidine dose was chosen according to previous studies from our laboratory, showing that these doses were adequate to inhibit central sympathetic activity and also to normalize arterial pressure in hypertensive animals (19). The treatment was performed by the introduction of the solution via a stainless-steel gavage needle (cannula diameter, 1.2 mm) attached to a syringe, and all procedures were performed at the same time of day. The normotensive control group consisted of WKY rats treated with vehicle (WKY+VEH) and SHR were divided into two groups of animals (minimum of 10 animals per group), treated with vehicle (SHR+VEH) or clonidine (SHR+CLO).

### Systolic blood pressure and heart rate

Systolic arterial pressure (SAP) and heart rate were measured in conscious animals using a computerized tail-cuff plethysmography system, a noninvasive blood pressure acquisition system (Visitech Blood Pressure Analysis System, model BP-200, USA). The rats were adapted to the pre-warmed tail-cuff apparatus for three consecutive days to acclimatize them and to avoid stress during the experimental procedures. Each session consisted of 15 cycles of measurements; the first 5 cycles were acclimation cycles and the data were not used, whereas the following cycles were used for the calculation of mean values of hemodynamic parameters.

### Quantification of total leukocytes

Total blood was collected in microtubes containing EDTA (0.5 mL Vacutube, Biocon, Brazil). The quantification of leukocytes was performed by impedance detection using an automated hematological Poch Analyzer 100-iV DIFF (Sysmex, Japan).

### Plasma and organ collection and peripheral blood leukocyte isolation

The animals were sacrificed with an anesthetic overdose (urethane 1.5 mg/kg, *ip*), the blood was collected by cardiac puncture, and the adrenal glands and the lower brainstem were removed for western blot analysis.

Leukocytes were obtained after centrifugation at 350 *g* at room temperature for 20 min of the whole blood followed by cycles of red blood cell lysis with a solution containing 0.144 M ammonium chloride and 17 mM Tris-HCl.

### Catecholamine measurements by ELISA

The measurement of plasma adrenaline and noradrenaline was performed using 2-CAT ELISA Fast TRACK^®^ (LDN Labor Diagnostika Nord GmbH & Co., Germany) according to the manufacturer's protocol. ELISA assay is widely used in experimental studies and accepted in clinical practice. Moreover, it has already been shown that the ELISA assay is accurate, sensitive, specific, and precise in the evaluation of plasma catecholamines (20). Linear regression analysis of adrenaline and noradrenaline concentrations measured with ELISA and with HPLC yield highly significant correlations (20).

### Flow cytometry

Leukocytes (10^6^) were processed for extracellular β_2_-AR and intracellular TH analysis. For TH evaluation, permeabilization was performed with 0.1% saponin. Nonspecific binding was blocked with inactivated goat serum at 4°C for 30 min and incubation with primary antibody (rabbit anti-tyrosine hydroxylase at 1:10 dilution, ab137869; Abcam, UK) or rabbit anti-receptor β_2_-adrenergic at dilution of 1:50 (ab137494; Abcam) for 20 min. After washes with phosphate buffered saline (PBS), the incubation with secondary antibody conjugated to Alexa Fluor 488 at 1:1000 dilution (A-21206; Invitrogen, USA) was performed for 20 min. The samples were fixed with 2% PFA, and analysis was carried out in the Cyan ADP Analyzer system (Beckman Coulter, Inc., USA). Biological samples were analyzed in a minimum of three technical replicates. The data were analyzed with Summit software version 4.3 (Beckman Coulter).

### Western blot analysis

Proteins were extracted from 100 mg tissue/mL lysis buffer [25 mM Tris-HCl; 150 mM sodium chloride, 1 mM EDTA; 50 mM Na fluoride; 1 mM Na orthovanadate; 1% triton x-100; 1 mM phenylmethylsulfonyl fluoride; 1/100 protease and phosphatase inhibitors cocktail (Sigma-Aldrich)]. The samples were sonicated twice and centrifuged at 12,000 *g* for 20 min at 4°C and the supernatant was kept frozen at -80°C. Proteins (50 µg/lane) were separated by SDS/PAGE (12%) and analyzed by immunoblotting with specific primary antibodies [rabbit anti-tyrosine hydroxylase at 1:5000 dilution (58 kDa) (ab137869; Abcam) or rabbit anti-receptor β_2_-adrenergic at 1:2000 dilution (46 kDa) (ab137494; Abcam)]. The immunogen utilized by the antibody manufacturer was a synthetic peptide encompassing a sequence within aa 340-413, which minimizes the cross-reactivity with different subtypes of β-ARs (β1, β2, and β3). For loading control, we used mouse anti-glyceraldehyde 3-phosphate dehydrogenase (GAPDH, 36 kDa) monoclonal antibody (10R-G109a; Fitzgerald Industries, USA). The membranes were incubated with secondary goat anti-mouse IgG HRP-labeled antibody (cat #31430; Thermo Scientific, Inc., USA) or with secondary goat anti-rabbit IgG HRP-labeled antibody (cat #31460; Thermo Scientific, Inc.) at 1:10000 dilution for 1 h at room temperature, followed by incubation with chemiluminescent kit Supersignal (Thermo Scientific, Inc.) and exposition to X-ray film. Biological samples were analyzed in a minimum of three technical replicates. The X-ray film bands densitometry analysis was performed with the software Image Studio Lite version 4.0 (LI-COR Biotechnology - UK Ltd, United Kingdom) and standardized by the GAPDH density. Considering that a unique band should be visible in western blot assays, it was technically impossible to determine β_2_-AR expression in the adrenal glands, since an extra band of 20 kDa was observed in the adrenal gland samples. Therefore, these data were not included in the results. Moreover, in the leukocytes, the small protein quantity obtained from the samples precluded the assessment of TH and β_2_-AR by western blot analysis with the available antibodies.

### Statistical analysis

Data are reported as means±SD or median (interquartile range). D'Agostino's K-squared test and Pearson's normality tests were used to evaluate normality of the data. Statistical analyses between two groups with normal distribution were performed using a two-tailed unpaired *t*-test, and if data did not present a normal distribution, a two-tailed Mann-Whitney test was used. One-way ANOVA followed by Tukey's *post hoc* test was employed in the analyses of more than two groups with normal distribution. Kruskal-Wallis test followed by Dunn's post-test was used in the evaluations among three groups with abnormal distributions. The Pearson correlation coefficient was used to measure the strength of the linear relationship. Differences with P values of less than 0.05 were considered significant. All analyses were performed using the statistical program GraphPad Prism 5 (GraphPad Software Inc., USA).

## Results

### Blood pressure, heart rate, and plasma catecholamine levels after chronic treatment with clonidine

As expected, hypertensive animals treated with vehicle (SHR+VEH) presented higher systolic blood pressure and heart rate than did normotensive WKY rats (P<0.001 and P<0.05, respectively; n=11-15, Table 1). Moreover, SHR+VEH showed increased plasma levels of adrenaline and noradrenaline compared with the normotensive animals (P<0.05 and P<0.01, respectively) (Table 1, n=11-15 animals/group).

**Table 1:** Systolic blood pressure, heart rate, plasma catecholamines, and total blood leukocyte quantification in normotensive animals (WKY) and spontaneously hypertensive rats (SHR) treated with vehicle (SHR+VEH) and hypertensive animals treated with clonidine (SHR+CLO).

	WKY	SHR+VEH	SHR+CLO
Systolic blood pressure (mmHg)	132±7	207±8***	129±12^###^
Heart rate (bpm)	363±22	393±31*	308±32***^###^
Plasma adrenaline (nM)	4.6 (1.3-5.1)	5.7 (5.0-5.5)*	3.9 (3.0-4.9)^#^
Plasma noradrenaline (nM)	51.2 (5.2-57.2)	68.8 (46.5-80.0)**	18.0 (6.0-52.5)^##^
Total number of leukocytes (x 10^3^/mm^3^)	8.0±3.3	8.1±1.9	8.7±3.0

Data are reported as means±SD for hemodynamic parameters and leucocyte quantification, or median (interquartile range) for catecholamine levels (n=11-15 animals/group). *P<0.05, **P<0.01, and ***P<0.001 *vs* WKY; ^#^P<0.05, ^##^P<0.01, and ^###^P<0.001 *vs* SHR+VEH [one-way ANOVA (hemodynamic measurement and leucocyte quantification) and Kruskal-Wallis test (catecholamine level)].

Compared to SHR+VEH, clonidine treatment reduced systolic pressure to values similar to those obtained in the normotensive group of WKY rats. Reduced heart rate was also observed in the clonidine-treated group, compared with both WKY (P<0.0001) and SHR+VEH (P<0.001). We observed significant reductions in plasma noradrenaline and adrenaline levels after 28 days of clonidine treatment compared with SHR+VEH (P<0.01 and P<0.01, respectively, Table 1).

### TH and β_2_-AR levels in adrenal glands and lower brainstem after clonidine treatment

**Figure 1:**
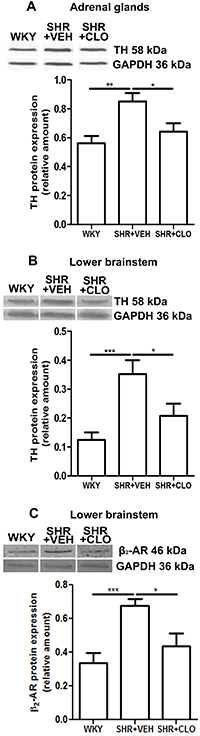
Protein expression levels of tyrosine hydroxylase (TH) in adrenal glands (**A**) and of TH (**B**) and β_2_-adrenergic receptor (β_2_-AR) expression (**C**) in the lower brainstem using western blot analysis. GAPDH was used as an endogenous control for the normalization of relative protein expression. Data are reported as means±SD for n=7-13 animals/group. *P<0.05, **P<0.001, and ***P<0.0001 (one-way ANOVA). WKY: normotensive Wistar Kyoto rats; SHR: spontaneously hypertensive rats; VEH: vehicle; CLO: clonidine.

In the adrenal gland, TH showed higher expression in SHR+VEH (0.85±0.17) than in WKY controls (0.56±0.16, P<0.01). Compared with non-treated SHR (0.85±0.17), clonidine treatment in SHR induced a significant reduction in TH levels in the adrenal glands (0.64±0.15, P<0.05) (Figure 1A).

In the brainstem, we found that TH (0.35±0.17) and β_2_-AR (0.68±0.13) were more highly expressed in the SHR+VEH group than in the normotensive control groups (0.12±0.09, P<0.001 and 0.33±0.19, P<0.001, respectively). Both protein levels in the brainstem were decreased in the clonidine group (0.21±0.14 and 0.44±0.24, respectively) compared with SHR+VEH (0.35±0.17, P<0.05 and 0.68±0.13, P<0.05, respectively) (Figure 1B and C).

### Effects of clonidine treatment on the levels of TH and β_2_-AR in leukocytes

The total leukocyte count was not different among the groups. The WKY group presented 8.03±3.31×10^3^ leukocytes/mm^3^, SHR+VEH had 8.14±1.95×10^3^ leukocytes/mm^3^, and SHR+CLO contained 8.72±2.97×10^3^ leukocytes/mm^3^ (Table 1).

**Figure 2:**
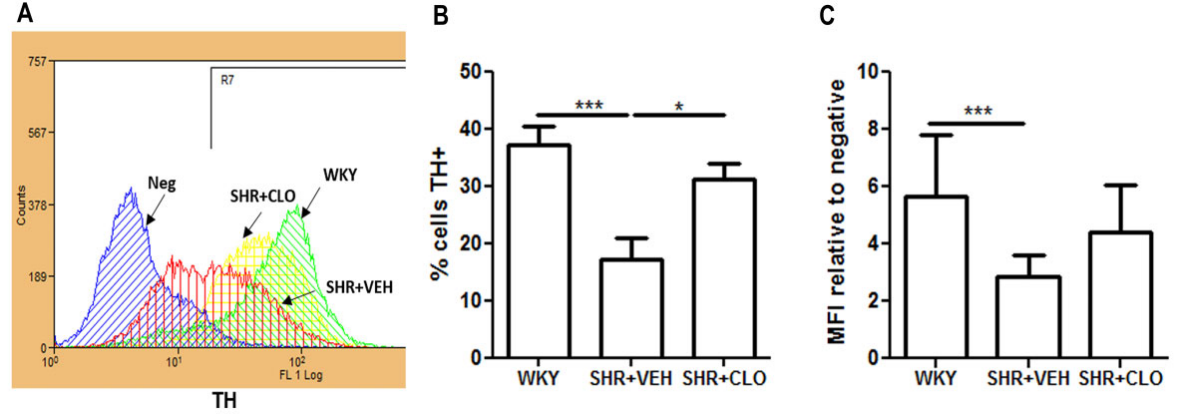
Representative histograms of tyrosine hydroxylase (TH) labeling (**A**). Quantification of the percentage of leukocytes expressing TH (**B**) and the median fluorescence intensity (MFI) (**C**) of TH labeling in total leukocytes from WKY, SHR+VEH, and SHR+CLO groups. Data are reported as means±SD for n=10 animals/group. *P <0.05 and ***P<0.0001 (one-way ANOVA). WKY: normotensive Wistar Kyoto rats; SHR: spontaneously hypertensive rats; VEH: vehicle; CLO: clonidine.

Flow cytometry analysis revealed significant differences in the percentage of positive measures for TH between WKY rats (37.1±11.3%) and SHR+VEH rats (17.1±14.5%, P<0.001) (Figure 2B, n=10 animals/group). Leukocytes from SHR+CLO presented a higher percentage of positive TH cells (31.1±7.9%) than those from SHR+VEH (17.1±14.5%, P<0.05), similar to the level in WKY rats (Figure 2A and B).

We observed that the median fluorescence intensity of TH was higher in the leukocytes of normotensive WKY rats (5.6±2.1) than in the leukocytes of SHR+VEH (2.8±0.7, P<0.001) (Figure 2C).

**Figure 3:**
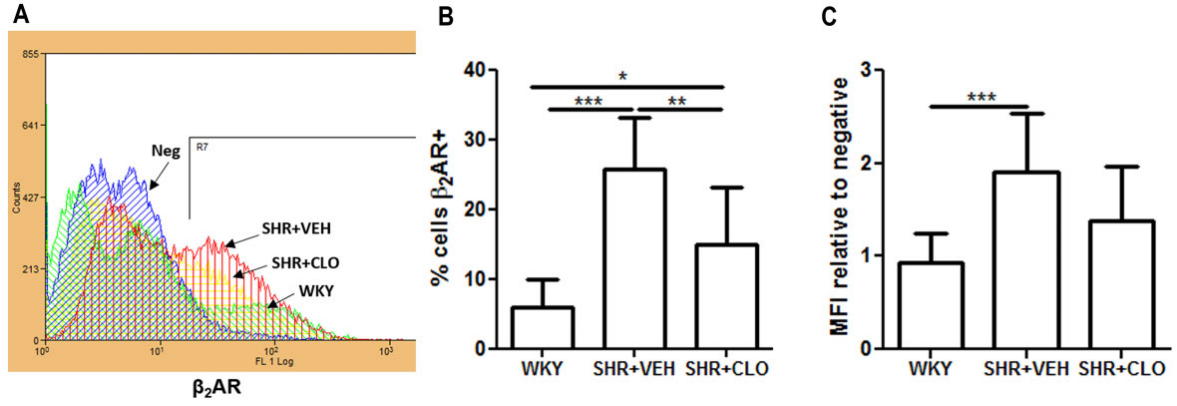
Representative histograms of β_2_-adrenergic receptor (β_2_-AR) labeling (**A**). Quantification of the percent of leukocytes expressing β_2_-AR (**B**) and the median fluorescence intensity (MFI) of the receptor (**C**) labeling in total leukocytes from WKY, SHR+VEH and SHR+CLO. Data are reported as means±SD for n=10 animals/group. *P<0.05, **P<0.01, and ***P<0.0001 (one-way ANOVA). WKY: normotensive Wistar Kyoto rats; SHR: spontaneously hypertensive rats; VEH: animals treated with vehicle; CLO: animals treated with clonidine.

A lower percentage of leukocytes positive for β_2_-AR was observed in WKY rats (6.0±3.9%) than in SHR+VEH (25.7±7.4%, P<0.001). Compared with SHR+VEH (25.7±7.4%, P<0.010), clonidine treatment resulted in a reduction in β_2_-AR-positive measures in leukocytes (15.0±8.1%). However, clonidine treatment induced a higher percentage of β_2_-AR-positive cells than was observed in normotensive WKY (6.0±3.9%, P<0.05) (Figure 3A and B).

We found that the median fluorescence intensity of β_2_-AR was lower in WKY leukocytes (0.94±0.30) than in SHR+VEH leukocytes (1.91±0.64, P<0.001) (Figure 3C).

### Correlation analysis

**Figure 4:**
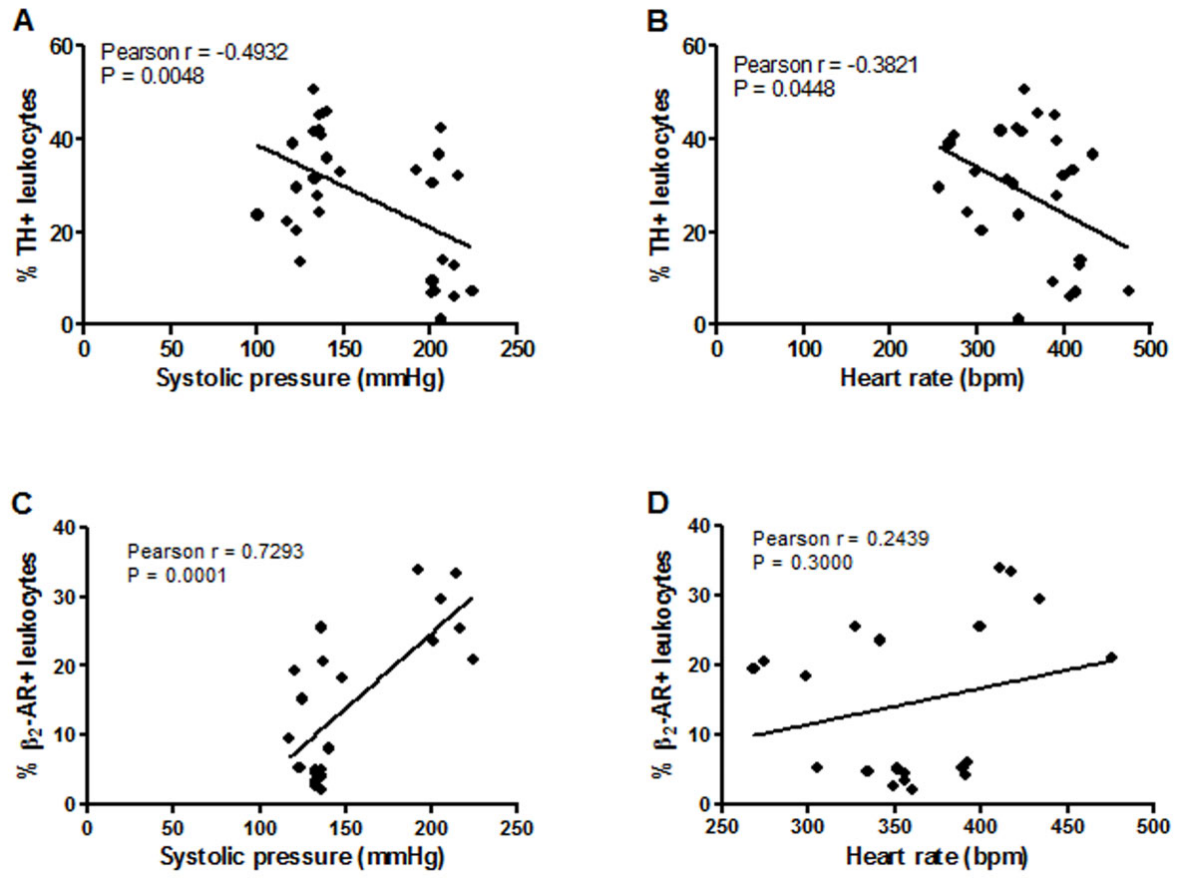
Pearson's correlation of the percentage of leukocytes positive for tyrosine hydroxylase (TH) (**A** and **B**) or β_2_-adrenergic receptor (β_2_-AR) (**C** and **D**) with heart rate and systolic blood pressure.

**Figure 5:**
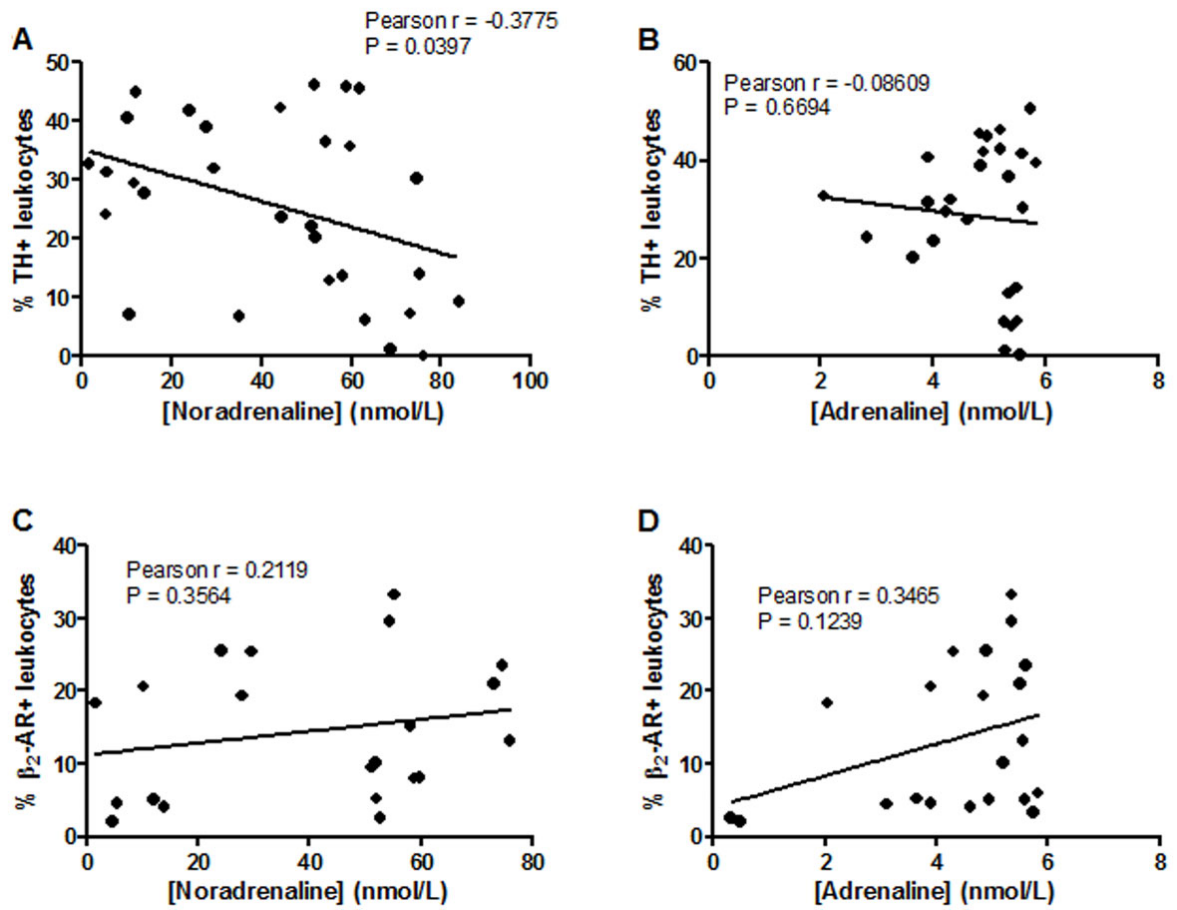
Pearson's correlation between the percentage of leukocytes expressing tyrosine hydroxylase (TH) (**A** and **B**) or β_2_-adrenergic receptor (**C** and **D**) (β_2_-AR) and plasmatic catecholamine levels.

The correlations of TH and β_2_-AR expression in leukocytes with hemodynamic parameters (Figure 4) and catecholamine levels (Figure 5) were evaluated. We observed inverse correlations between the percentage of leukocytes expressing TH with both systolic pressure (r=-0.4-932; P=0.0048) (Figure 4A) and heart rate (r=-0.3821; P=0.0448) (Figure 4B). There was a direct correlation between β_2_-AR expression in leukocytes and systolic pressure (r=0.7293; P=0.0001) (Figure 4C), which was not observed between receptor expression and heart rate (Figure 4D).

There was an inverse linear relationship (r=-0.3775; P=0.0397) between the percentage of leucocytes positive for TH and plasma noradrenaline levels evaluated in normotensive and hypertensive animals after long-term treatment with clonidine or vehicle (Figure 5A). There were also statistically significant correlations when we considered only the group of hypertensive animals as follows: percentage of leucocytes positive for TH *vs* systolic arterial pressure (r=-0.4571 and P=0.0372) and *vs* heart rate (r=-0.5176 and P=0.0163); percentage of leucocytes positive for β_2_-AR *vs* systolic arterial pressure (r=0.6217 and P=0.0412) and heart rate (r=0.4702 and P=0.1230); percentage of leucocytes positive for TH *vs* plasma noradrenaline concentrations (r=-0.5226 and P=0.0151).

## Discussion

It is recognized that sympathetic hyperactivity is a therapeutic target in the management of arterial hypertension (21), not only through the use of drugs such as beta-blockers or centrally acting antihypertensives but also through interventions such as baroreflex activation (21) and renal denervation (22). These latter procedures are somewhat recent, and further studies should be conducted to better elucidate the controversies regarding the efficacy and mechanisms of these interventions.

However, the assessment of sympathetic activity in patients is performed using methods with low sensitivity or that are difficult to implement in clinical practice (23), including plasma and/or urine catecholamine levels, postganglionic sympathetic neural traffic with microneurography, noradrenaline spillover, or heart rate spectral power analyses (23,24).

Plasma and urine catecholamine measurements are noninvasive and are the most commonly used techniques to evaluate sympathetic activity in clinical practice. However, these methods depend on the levels of secretion, tissue clearance, and re-uptake of catecholamines. For instance, it has already been demonstrated that plasma noradrenaline levels correspond to noradrenaline concentrations released from presynaptic neurons and, consequently, a sympathetic nerve activity marker, only when norepinephrine re-uptake transporter is blocked (25). Moreover, it is important to note that catecholamines found in the plasma are also released by other sites than sympathetic nerves and adrenal glands; actually, catecholamines can also be released by platelets (13) and endothelial cells (12). Finally, the plasma concentrations of dopamine have already been shown to be independent of sympathetic activity in cardiovascular diseases, including arterial hypertension (26). Moreover, platelet levels of free-catecholamines are not considered to be a marker of increased sympathetic activity in essential hypertension (27). In general, dopamine is not taken into account in the evaluation of sympathetic overactivity in arterial hypertension, either in experimental models or in the clinical setting. Thus, we estimated that it was not crucial to evaluate plasma levels of dopamine in the present study.

Microneurography is the recording of postganglionic muscle sympathetic nerve activity, usually studying peroneal or brachial nerves. These local evaluations are invasive and complex to perform in the clinical setting (28). Another method to evaluate sympathetic activity is the measurement of the radioactive noradrenaline appearance rate in plasma, which was correlated with muscle sympathetic activity (29). Nevertheless, the technique includes radioactivity exposure risk and accesses sympathetic nerves of internal organs (23).

Therefore, it is important to search for new markers of systemic sympathetic activity. In this context, we analyzed the expression of TH and β_2_-AR in peripheral blood leukocytes and the relationship between the expression of these proteins in the adrenal gland or lower brainstem and systolic blood pressure.

TH appears to be a key component in the development of hypertension in SHR, since higher TH mRNA levels are detected in the adrenal gland and medulla oblongata of SHR than in those of WKY (30). Moreover, alterations in the ubiquitin-proteasome system in hypothalamic and brainstem neurons from SHR have already been demonstrated, which could explain the higher levels of TH in these animals (31).

Additionally, β-AR is present in the CNS, and the intracerebroventricular administration of β-AR nonselective antagonists prevents the increase in renal nerve activity induced by stress in SHR (32). In addition, the intracerebroventricular, intrahypothalamic, or intracisternal administration of a β_2_-AR selective antagonist also prevented the increase in arterial pressure induced by C_1_ region electrical stimulation (33).

Our results confirmed the presence of hyperactivity of the SNS in arterial hypertension, as demonstrated by increased TH and β_2_-AR expression in the lower brainstem and in the adrenal gland of SHR compared with WKY. Additionally, the chronic treatment of the animals with clonidine, a centrally acting sympathoinhibitory drug, resulted in a reduction in the expression of both TH and β_2_-AR, thus confirming the association between sympathetic activity and the expression of these proteins. Importantly, there was a positive and significant correlation between the expression of these proteins and systolic blood pressure. Finally, hypertensive animals presented higher adrenaline and noradrenaline plasma levels. Taken together, the above-mentioned results could indicate a down-regulation or desensitization of β_2_-AR in the leukocytes resulting from the chronic elevation of plasma catecholamine levels in the experimental model of primary arterial hypertension used in the present study (SHR). In this context, it has already been demonstrated that chronic catecholamine elevation induces a decrease of the number of plasma membrane β_2_-AR in lymphocytes. This phenomenon was shown to be reversible upon normalization of plasma catecholamine levels in patients with pheochromocytoma (34). Nevertheless, in the present work, we did not evaluate function but only expression of the β_2_-AR in the leukocytes.

Previous studies demonstrated that mononuclear leukocytes synthesize catecholamines and express β_2_-AR (35). In the present study, we investigated the expression of TH and β_2_-AR in total leukocytes using flow cytometry, and showed that leukocytes from both WKY and SHR expressed TH and β_2_-AR. It is important to note that platelets were eliminated from the analysis in the side and forward scatter parameters of the flow cytometry assays by the different sizes of these cells; moreover, circulating endothelial cells are quite rare in peripheral blood, as described previously (36). Thus, both cell types, which are known to release catecholamines, do not appear to have contributed to our results.

Considering the groups of normotensive and hypertensive animals as a single population, we found an inverse correlation for TH expression and a direct correlation for β_2_-AR expression in leukocytes with systolic blood pressure. There was also a higher number of leukocytes positive for TH in normotensive animals than in hypertensive animals. On the other hand, a lower percentage of leukocytes presented β_2_-AR in normotensive animals; therefore, higher levels of leukocytes positive for the receptor were present in animals with higher systolic blood pressure values.

The use of biomarkers with a negative correlation with the biological process has been previously proposed (37). In this context, Demler et al. (38) demonstrated that biomarkers with negative correlations present better power for risk prediction, using area under the receiver operating characteristic curve (ROC) as a measure of evaluating the quality of risk prediction.

Our results corroborate data from the literature (18), demonstrating that mononuclear cells from hypertensive patients present higher expression of β_2_-AR than those from normotensive controls. In that study, a significant correlation of β_2_-AR density in mononuclear cells with mean arterial pressure was also found. This analysis was performed by grouping, as a single population, the data of normotensive patients together with that of hypertensive patients (18). In addition to the alterations in β_2_-AR receptor expression, alterations in α_1_-adrenergic receptor expression were observed in peripheral blood lymphocytes from patients with essential hypertension and from the animal model of primary hypertension of the SHR (16). It was also observed that patients with heart failure and severe depression had higher β-AR activity in mononuclear cells (39).

Our results on the expression of leukocytes positive for TH and β_2_-AR did not exclude a possible direct effect of clonidine on these cells. To evaluate this effect, *in vitro* analyses of leukocyte treatment with clonidine should be performed. However, the incubation of peripheral blood mononuclear cells with 4 μM clonidine did not alter the density of β-AR without distinction between β_1_- and β_2_-AR (40), indicating that clonidine had no direct effect on these cells.

The causes and the biological significance of the relative reduction of TH expression in the peripheral blood leukocytes of hypertensive animals, compared to the normotensive counterpart, remain to be elucidated. This could be related to a counter-regulatory mechanism resulting from the increased plasma levels of catecholamines in hypertensive animals.

In summary, our results demonstrated that the percentage of leukocytes expressing TH and β_2_-AR was altered in arterial hypertension and can be modulated by central sympathetic inhibition with clonidine treatment, thus suggesting that the expression of these proteins could be candidates for the development of peripheral markers of the central sympathetic activity in primary arterial hypertension.
